# The 2025 Los Angeles Wildfires and Outpatient Acute Health Care Utilization

**DOI:** 10.1001/jamahealthforum.2025.4632

**Published:** 2025-11-26

**Authors:** Joan A. Casey, Yuqian M. Gu, Lara Schwarz, Timothy B. Frankland, Lauren B. Wilner, Heather McBrien, Nina M. Flores, Arnab K. Dey, Gina S. Lee, Chen Chen, Tarik Benmarhnia, Sara Y. Tartof

**Affiliations:** 1 Department of Environmental and Occupational Health Sciences, University of Washington School of Public Health, Seattle; 2 Department of Epidemiology, University of Washington School of Public Health, Seattle; 3 Department of Research & Evaluation, Kaiser Permanente Southern California; 4 Division of Environmental Health Sciences, University of California, Berkeley School of Public Health; 5 Kaiser Permanente Hawaii Center for Integrated Health Care Research; 6 Department of Environmental Health Sciences, Columbia University School of Public Health; 7 Scripps Institution of Oceanography, University of California, San Diego; 8 Irset Institut de Recherche en Santé, Environnement et Travail, UMR-S 1085, Inserm, University of Rennes, EHESP, Rennes, France

## Abstract

**Importance:**

January 2025 brought devastating wildfires to Los Angeles (LA) County, California, causing poor air quality, destroying homes and businesses, and displacing thousands of people.

**Objective:**

To promptly determine if the January 2025 LA fires increased outpatient acute health care utilization.

**Design, Setting, and Participants:**

This cohort study assessed electronic health care records of 3.7 million members of Kaiser Permanente Southern California (KPSC) who met enrollment criteria. Of those, 305 258 were highly exposed and 1.4 million were moderately exposed. KPSC serves 4.7 million people across 10 counties. Analyses were conducted January-June 2025.

**Exposures:**

Highly exposed members were those who resided in a census tract within 20 km of burn zones, and moderately exposed members were those who resided in tracts that were 20 km or more miles from the burn zones but within LA County. The maximum wildfire burn zone reached by an LA or Ventura county wildfire by January 17, 2025 was used.

**Main Outcomes and Measures:**

Daily outpatient and virtual acute care visits in 5 categories: all-cause (or any medical reason), cardiovascular, injury, neuropsychiatric, and respiratory were identified and analyzed by a 2-stage interrupted time-series model using machine-learning algorithms to determine if and by how much the January 2025 LA fires increased outpatient acute health care utilization.

**Results:**

Across the week following the January 7 LA fires’ ignition, there were excess visits from the 3.7 million study population (1.94 million women [52.2%], 1.77 million men [47.7%]; median age, 42 years [IQR, 21-62]; 11.5% Asian, 7.8% Black, 43.8% Hispanic, and 29% White). Virtual respiratory visits were 42% (95% empirical CI, 23%-60%) higher than expected in highly exposed groups and 36% (95% empirical CI, 19%-54%) higher than expected in moderately exposed groups. Similarly, the highly exposed and moderately exposed groups had 44% and 40% more than expected virtual cardiovascular visits, respectively, and 27% and 31% more than expected outpatient respiratory visits, respectively (n=2866 and n=5166 total excess visits). All-cause virtual care visits were also elevated in the moderately exposed group, potentially representing displaced in-person care. Among highly exposed members, outpatient and virtual injury visits and outpatient neuropsychiatric visits were 18% higher than expected on January 7. Extrapolating to all insured LA County residents, an excess of 15 792 cardiovascular and 18 489 respiratory virtual care visits and 27 903 respiratory outpatient visits was estimated during the week following ignition.

**Conclusions and Relevance:**

This cohort study observed substantial increases in acute health care utilization, especially virtual care-seeking following the LA fires. As disruptive climate events increase, such data are essential to inform health care preparedness and response.

## Key Points

**Question:**

Did acute outpatient and virtual care visits increase more than expected among Kaiser Permanente Southern California members following the 2025 Los Angeles fires?

**Findings:**

There were 8032 excess outpatient respiratory visits and 3375 excess virtual cardiovascular and respiratory visits, representing 27% to 44% higher than expected visits among the highly exposed (<20 km from the burn zone) and moderately exposed groups (LA County, 20 km from burn zone) exposed over the week following the January 7, 2025, LA fires’ ignition. Outpatient and virtual injury and neuropsychiatric visits were also elevated at the same time.

**Meaning:**

Substantial increases in outpatient and virtual health care utilization following the LA fires suggest emerging importance of virtual care during disruptive climate events.

## Teaser

This cohort study assesses the January 2025 LA fires’ impact on acute outpatient health care use at Kaiser Permanente Southern California.

## Introduction

On January 7, 2025, the Palisades and Eaton fires ignited in Los Angeles (LA) County, California, followed by other fires in LA and neighboring Ventura County. Fueled by climate change and strong Santa Ana winds, the fires burned more than 38 000 acres and destroyed more than 16 000 structures.^1^ Direct fatalities totaled 29,^2^ but the true health burden will exceed that tally. Smoke caused fine particulate matter (PM_2.5_) concentrations to exceed the US Environmental Protection Agency 24-hour standard of 35 μg/m^3^ for several days and briefly topped 400 μg/m^3^ in LA on January 8 before returning to background levels by January 12.^3^

Previous studies have identified short-term wildfire PM_2.5_ and wildfire burn zone proximity as risk factors for mortality and adverse cardiorespiratory, mental health, and injury outcomes.^4,5,6^

Herein, we rapidly assessed the January 2025 LA fires’ impact on acute outpatient health care utilization at Kaiser Permanente Southern California (KPSC).

## Methods

KPSC is an integrated health care system serving more than 4.7 million individuals. Its electronic health record (EHR) database contains longitudinal patient residential addresses, sociodemographic characteristics, and diagnoses across all care settings. This study included all KPSC members from November 1, 2022, to January 21, 2025 (eFigure 1 in Supplement 1). This study followed the Strengthening the Reporting of Observational Studies in Epidemiology (STROBE) guidelines, and the protocol was approved by the KPSC and WIRB–Copernicus Group institutional review boards, which waived the requirement for informed consent.

We classified census tracts a priori into 3 exposure levels based on their proximity to the maximum burn zone of 7 LA-area wildfires: Auto, Eaton, Hurst, Kenneth, Lidia, Palisades, and Sunset.^2^ Highly exposed members resided in census tracts within 20 km from a wildfire burn zone, and moderately exposed members lived in tracts 20 km or more away but within LA County (eMethods 1 and eFigure 2 in Supplement 1). By assessing exposure via residential proximity to the burn zones, we aimed to capture varying levels of reduced air quality, psychosocial stress, and community disruption from the LA fires.

We identified daily virtual and outpatient visits in 5 disease categories using *International Classification of Diseases*, *Tenth Revision,* codes (all-cause, cardiovascular [I00-I99], injury [S00-T88], neuropsychiatric [F01-F99], and respiratory [J00-J99]). We aggregated visits by type, cause, day, and wildfire exposure category.

Time-varying census tract-level covariates included daily maximum and minimum temperature and humidity, wind velocity, and surface downward shortwave radiation processed from 4-km^2^ resolution Gridded Surface Meteorological (gridMET) data, as well as weekly wastewater surveillance data on influenza, respiratory syncytial virus, and SARS-CoV-2 from the LA County Department of Public Health.

We used a novel 2-stage interrupted time-series (ITS) analysis^7^ to assess the relationship between the LA fires and daily outpatient acute care utilization in the week following the January 7 LA fires’ ignition. Briefly, this 2-stage ITS analysis was coupled with hybrid machine learning algorithms (eg, Prophet-Extreme Gradient Boosting) to optimize the prediction of counterfactual trends (acute care utilization in the absence of the LA fires), and the optimal parameter set was determined by minimizing the root mean square error across cross-validation folds (eMethods 2 in Supplement 1). This approach may reduce bias present in traditional estimates.^8^

In 2 sensitivity analyses, we first assessed changes in respiratory visits among minimally exposed members who lived in tracts 20 km or more from wildfire burn zones in 9 non-LA KPSC catchment counties (eMethods 1 in Supplement 1). Then we evaluated an alternative buffer distance (<10 km) to define highly exposed census tracts.

Analyses were conducted using R Statistical Software version 4.4.1 and Python version 3.12.2.

## Results

**Figure 1.**
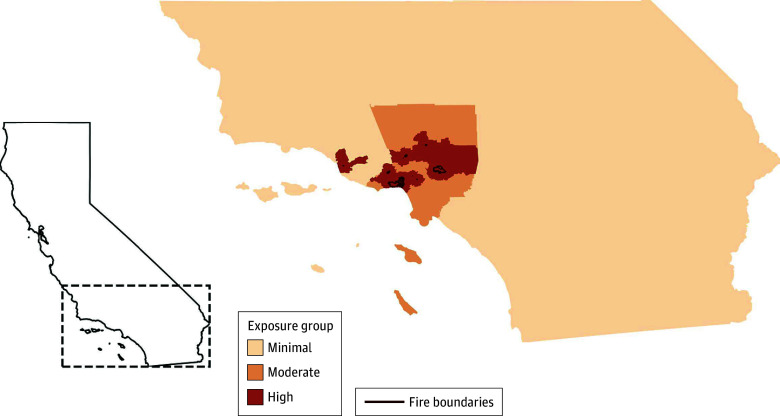
The January 2025 Los Angeles Wildfires Overlaid With Highly, Moderately, and Minimally Exposed Census Tracts The 3 exposure categories included: (1) highly exposed census tracts within 20 km of a wildfire burn zone; (2) moderately exposed census tracts more than 20 km from a wildfire burn zone but within Los Angeles (LA) County; (3) minimally exposed census tracts outside LA County but within the Kaiser Permanente Southern California catchment area. The map depicts the maximum wildfire burn zone reached by the 7 LA wildfires as of January 17, 2025.

The study population consisted of 3.7 million KPSC members (1.94 million women [52.2%], 1.77 million men [47.7%]; median age, 42 years [IQR, 21-62]; 11.5% Asian, 7.8% Black, 43.8% Hispanic, and 29% White; Table), of whom 305 258 were highly exposed (Figure 1), accounting for 13.6% of all insured LA residents living within 20 km of an LA fire burn zone.

**Table.** Characteristics of the Kaiser Permanente Southern California Study Population, November 2022-January 2025

	LA fires exposure, No. (%)			
	Total (N = 3 717 210)	Type of exposure^a^		
		High (n = 305 258)	Moderate (n = 1 373 419)	Minimal (n = 2 038 533)
Age, median (IQR), y^b^	42 (21-62)	46 (26-65)	42 (22-61)	42 (21-61)
Sex				
Female	1 941 970 (52.2)	158 011 (51.8)	723 153 (52.7)	1 060 806 (52.0)
Male	1 774 871 (47.7)	147 202 (48.2)	650 138 (47.3)	977 531 (48.0)
Other	167 (<0.1)	22 (<0.1)	68 (<0.1)	77 (0)
Unknown	202 (<0.1)	23 (<0.1)	60 (<0.1)	119 (0)
Race and ethnicity^c^				
Hispanic	1 627 406 (43.8)	98 493 (32.3)	719 131 (52.4)	809 782 (39.7)
Non-Hispanic Asian	426 054 (11.5)	118 389 (38.8)	160 361 (11.7)	221 815 (10.9)
Non-Hispanic Black	290 701 (7.8)	43 878 (14.4)	158 081 (11.5)	117 106 (5.7)
Non-Hispanic White	1 078 707 (29.0)	15 514 (5.1)	231 239 (16.8)	729 079 (35.8)
Unknown	173 378 (4.7)	16 137 (5.3)	60 910 (4.4)	96 331 (4.7)
Other	120 964 (3.3)	12 847 (4.2)	43 697 (3.2)	64 420 (3.2)

^a^ Based on the maximum wildfire burn zone reached by a Los Angeles (LA) County or Ventura County wildfire as of January 17, 2025, highly exposed members resided in a census tract within 20 km, moderately exposed members lived in tracts 20 km or more but within LA County, and minimally exposed members lived in tracts 20 km or more in other Kaiser Permanente Southern California (KPSC) catchment counties (Imperial, Kern, Orange, Riverside, San Bernardino, San Diego, San Luis Obispo, Santa Barbara, and Ventura).

^b^ Age was calculated as of January 7, 2025, or death.

^c^ Race and ethnicity were self-reported by KPSC members. Other includes individuals of Native American and Alaska Native, Pacific Islander, multiple races, and any other race not described.

**Figure 2.**
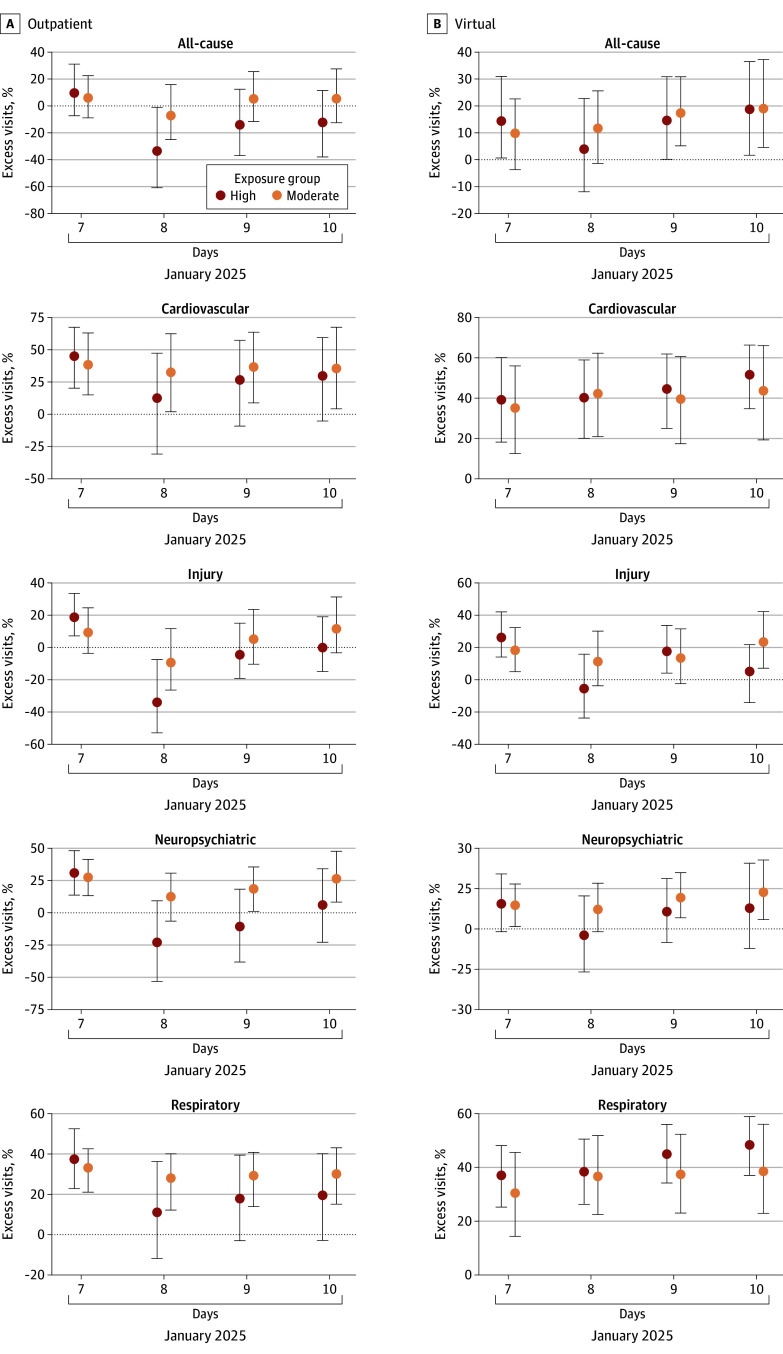
Estimated Change in the Percent of Patient Care Visits at Kaiser Permanente Southern California in the 4 Days Following the January 7, 2025, Ignition of the Los Angeles County Fires We used the maximum wildfire burn zone reached by a Los Angeles (LA) or Ventura County wildfire as of January 17, 2025, to define exposure. Highly exposed members resided in a census tract located within 20 km of burn zones, and moderately exposed members lived in tracts 20 km or more but within LA County. Results from an interrupted time-series model using Kaiser Permanente Southern California electronic health record data from November 1, 2022, to January 21, 2025, with daily maximum and minimum temperature and humidity, wind velocity, and surface downward shortwave radiation and weekly wastewater surveillance data on levels of 3 respiratory viruses as covariates. We employed a Monte Carlo simulation approach, performing 1000 model iterations to estimate 95% empirical CIs for the predictions. The data markers indicate point estimates for daily percent excess visits; whiskers, 95% empirical CIs.

Across the week following the January 7 LA fires’ ignition, virtual respiratory visits were higher than expected among highly exposed groups (42%; 95% empirical CI, 23%-60%) and among moderately exposed groups (36%; 95% empirical CI, 19%-54%), totaling 3375 excess visits (Figure 2; eTables 1 and 2 in Supplement 1). Generally, daily CIs were wide on the weekend of January 11 and 12 . Similarly, virtual cardiovascular visits were higher than expected: 44% in the highly exposed group and 40% in the moderately exposed group as were outpatient respiratory visits: 27% and 31%, respectively (n = 2866 and n = 5166 total excess visits). By applying these estimates to all insured LA County residents, an estimated 15 792 excess cardiovascular and 18 489 excess respiratory virtual care visits and 27 903 excess outpatient respiratory visits occurred during the week following ignition (eMethods 3 in Supplement 1).

On January 7, outpatient injury visits were 18% (n = 54, 95% empirical CI, 21%-97%), higher than expected in the highly exposed group, and injury virtual visits were higher than expected in both groups, 26% among the highly exposed and 18% among the moderately exposed groups (Figure 2; eTables 1-4 in Supplement 1). Outpatient neuropsychiatric visits were also higher than expected on January 7: 31% in the highly exposed group and 28% in the moderately exposed group totaling 330 excess visits (95% empirical CI, 147-512) among the highly exposed and 1263 (95% empirical CI, 611-1888) among the moderately exposed groups.

As with cardiovascular and respiratory visits, the moderately exposed group demonstrated several days with elevated outpatient injury and neuropsychiatric visits during the week following ignition, as well as a 19% exceedance of neuropsychiatric and all-cause virtual visits across the week.

In a sensitivity analysis, respiratory-related virtual visits in the minimally exposed group were 31% (95% empirical CI, 18%-45%) higher than expected during the week following January 7. When considering only tracts within 10 km from a burn zone as highly exposed, results remained similar (eFigures 3 and 4 in Supplement 1), but from January 7 through 9, we observed 31% (95% empirical CI, 12%-51%) higher than expected virtual injury visits, a greater increase over a longer period than in the primary analysis using an exposure definition of less than 20 km.

## Discussion

The LA fires ignited on January 7, 2025, and evacuations, power outages, displacement, and devastating loss of property and lives ensued.^2^ Over the following week, we observed elevated virtual and outpatient cardiovascular- and respiratory-related care-seeking among highly- and moderately-exposed KPSC members. Injury and neuropsychiatric care-seeking also increased, but less uniformly.

These findings align with research showing increased acute emergency and inpatient cardiorespiratory and mental health care use after wildfire smoke exposure.^5,9,10^ Studies of wildfire disasters have also documented increased injuries, like motor vehicle crashes during evacuation.^11^

Virtual care seeking increased more substantially than in-person care seeking following the LA Fires, suggesting health care systems should prioritize adequate virtual care staffing during climate events.^12^ Increased virtual care seeking likely reflects displaced in-person care, patient preferences, and increased illness. We saw potential catch-up outpatient care on January 13, after the worst of the smoke had cleared, but also instances of excess outpatient care in the week following ignition.

### Limitations

This study had limitations. Smoke and burn zone exposure was not differentiated. Both exposures differentially impact members of persistently disadvantaged groups.^13,14^ Early reports indicate many LA fires victims were older adults and disabled people.^15^ This time-series analyses did not assess vulnerability factors, such as housing quality or who successfully evacuated, and only considered changes in utilization during the week following the LA fires’ ignition. Future work should examine effect modification by individual and community characteristics and analyze longer-term health consequences, including disease incidence and morbidity displacement. Because it takes KPSC longer to reconcile cause-specific emergency department and inpatient visit data accurately, this initial study was restricted to outpatient and virtual visits. Finally, the minimally exposed respiratory virtual visit model had relatively poor performance and used LA County wastewater surveillance data, despite these tracts being outside LA. Given these limitations, we recommend cautious interpretation of the minimally exposed respiratory-related findings.

## Conclusions

Climate hazards will increasingly impact US cities. This study demonstrated that EHR data can rapidly inform health care delivery needs and population health impacts after such events. Substantial increases in acute care visits, especially virtual care seeking were identified, which will likely play a growing role in serving patients during disruptive climate events.

## Notes

Supplement 1. **eMethods 1.** Exposure assessment and confounding variables **eMethods 2.** The 2-stage interrupted time-series design **eMethods 3.** Extrapolating excess visit counts to all insured LA County residents **eFigure 1.** Flow diagram depicting exclusion criteria for members of the Kaiser Permanente Southern California study population, 2022-2025 **eFigure 2.** US Environmental Protection Agency monitoring station locations and station-level all-source daily PM_2.5_ concentrations during the week following the LA Fires **eFigure 3.** Estimated change in the percent of outpatient and virtual acute care visits **eFigure 4.** Estimated change in the count of outpatient and virtual acute care visits for all-cause, cardiovascular, injury, neuropsychiatric, and respiratory endpoints among Kaiser Permanente Southern California members **eTable 1.** Estimated change in the percent and count of virtual acute care visits for all-cause, cardiovascular, injury, neuropsychiatric, and respiratory endpoints among Kaiser Permanente Southern California highly-exposed members **eTable 2.** Estimated change in the percent and count of virtual acute care visits for all-cause, cardiovascular, injury, neuropsychiatric, and respiratory endpoints among Kaiser Permanente Southern California moderately exposed members **eTable 3.** Estimated change in the percent and count of outpatient acute care visits for all-cause, cardiovascular, injury, neuropsychiatric, and respiratory endpoints among Kaiser Permanente Southern California highly exposed members **eTable 4.** Estimated change in the percent and count of outpatient acute care visits for all-cause, cardiovascular, injury, neuropsychiatric, and respiratory endpoints among Kaiser Permanente Southern California moderately-exposed members
eReferences

Supplement 2.
Data Sharing Statement
