# Supplementary material for: The 2025 Los Angeles Wildfires and Outpatient Acute Health Care Utilization
Source: JAMA Health Forum. 2025 Nov 26;6(11):e254632. doi: 10.1001/jamahealthforum.2025.4632 (PMC12658665; doi:10.1001/jamahealthforum.2025.4632)
Supplement: Supplement 1. — eMethods 1. Exposure assessment and confounding variables eMethods 2. The 2-stage interrupted time-series design eMethods 3. Extrapolating excess visit counts to all insured LA County residents eFigure 1. Flow diagram depicting exclusion criteria for members of the Kaiser Permanente Southern California study population, 2022-2025 eFigure 2. US Environmental Protection Agency monitoring station locations and station-level all-source daily PM2.5 concentrations during the week following the LA Fires eFigure 3. Estimated change in the percent of outpatient and virtual acute care visits eFigure 4. Estimated change in the count of outpatient and virtual acute care visits for all-cause, cardiovascular, injury, neuropsychiatric, and respiratory endpoints among Kaiser Permanente Southern California members eTable 1. Estimated change in the percent and count of virtual acute care visits for all-cause, cardiovascular, injury, neuropsychiatric, and respiratory endpoints among Kaiser Permanente Southern California highly-exposed members eTable 2. Estimated change in the percent and count of virtual acute care visits for all-cause, cardiovascular, injury, neuropsychiatric, and respiratory endpoints among Kaiser Permanente Southern California moderately exposed members eTable 3. Estimated change in the percent and count of outpatient acute care visits for all-cause, cardiovascular, injury, neuropsychiatric, and respiratory endpoints among Kaiser Permanente Southern California highly exposed members eTable 4. Estimated change in the percent and count of outpatient acute care visits for all-cause, cardiovascular, injury, neuropsychiatric, and respiratory endpoints among Kaiser Permanente Southern California moderately-exposed members eReferences [file jamahealthforum-e254632-s001.pdf]

Supplemental Online Content

Casey JA, Gu YM, Schwarz L, et al. The 2025 Los Angeles wildfires and outpatient acute health care utilization. *JAMA Health Forum*. Published online November 26, 2025. doi:10.1001/jamahealthforum.2025.4632

eMethods 1. Exposure assessment and confounding variables

eMethods 2. The 2-stage interrupted time-series design

eMethods 3. Extrapolating excess visit counts to all insured LA County residents

eFigure 1. Flow diagram depicting exclusion criteria for members of the KPSC study population, 2022-2025

eFigure 2. US Environmental Protection Agency monitoring station locations and station-level all-source daily $PM_{2.5}$ concentrations during the week following the LA Fires

eFigure 3. Estimated change in the percent of outpatient and virtual acute care visits

eFigure 4. Estimated change in the count of outpatient and virtual acute care visits for all-cause, cardiovascular, injury, neuropsychiatric, and respiratory endpoints among Kaiser Permanente Southern California members

eTable 1. Estimated change in the percent and count of virtual acute care visits for all-cause, cardiovascular, injury, neuropsychiatric, and respiratory endpoints among Kaiser Permanente Southern California highly-exposed members

eTable 2. Estimated change in the percent and count of virtual acute care visits for all-cause, cardiovascular, injury, neuropsychiatric, and respiratory endpoints among Kaiser Permanente Southern California moderatelyexposed members

eTable 3. Estimated change in the percent and count of outpatient acute care visits for all-cause, cardiovascular, injury, neuropsychiatric, and respiratory endpoints among Kaiser Permanente Southern California highly exposed members

eTable 4. Estimated change in the percent and count of outpatient acute care visits for all-cause, cardiovascular, injury, neuropsychiatric, and respiratory endpoints among Kaiser Permanente Southern California moderately-exposed members

eReferences

This supplemental material has been provided by the authors to give readers additional information about their work.

© 2025 Casey JA et al. JAMA Health Forum.

TABLE OF CONTENTS

Materials and Methods

eMethods 1. Exposure assessment and confounding variables…………………………….................................... 2
eMethods 2. The 2-stage interrupted time-series design……………………..……….. 2
eMethods 3. Extrapolating excess visit counts to all insured LA County residents …………….......................... 4
eFigure 1. Flow diagram depicting exclusion criteria for members of the KPSC study population, 2022-2025......... 6
eFigure 2. (A) US Environmental Protection Agency monitoring station locations by geographic region, and (B) Station-level all-source daily $PM_{2.5}$ concentrations during the week following the LA Fires' ignition grouped by geographic region..7
eFigure 3. Estimated change in the percent of outpatient and virtual acute care visits for all-cause, cardiovascular, injury, neuropsychiatric, and respiratory endpoints among Kaiser Permanente Southern California (KPSC) members residing in census tracts <10km (sensitivity analysis) and <20km (primary analysis) of a burn zone in the week following the January 7, 2025 ignition of the LA Fires...
eFigure 4. Estimated change in the count of outpatient and virtual acute care visits for all-cause, cardiovascular, injury, neuropsychiatric, and respiratory endpoints among Kaiser Permanente Southern California (KPSC) members residing in census tracts <10km (sensitivity analysis) and <20km (primary analysis) of a burn zone in the week following the January 7, 2025 ignition of the LA Fires...

Results

eTable 1. Estimated change in the percent and count of virtual acute care visits for all-cause, cardiovascular, injury, neuropsychiatric, and respiratory endpoints among Kaiser Permanente Southern California (KPSC) highly-exposed members (n=305,258) in the week following the January 7, 2025 ignition of the LA Fires.. 8
eTable 2. Estimated change in the percent and count of virtual acute care visits for all-cause, cardiovascular, injury, neuropsychiatric, and respiratory endpoints among Kaiser Permanente Southern California (KPSC) moderately-exposed members (n=1,373,419) in the week following the January 7, 2025 ignition of the LA Fires........................ 9
eTable 3. Estimated change in the percent and count of outpatient acute care visits for all-cause, cardiovascular, injury, neuropsychiatric, and respiratory endpoints among Kaiser Permanente Southern California (KPSC) highly-exposed members (n=305,258) in the week following the January 7, 2025 ignition of the LA Fires......................... 10
eTable 4. Estimated change in the percent and count of outpatient acute care visits for all-cause, cardiovascular, injury, neuropsychiatric, and respiratory endpoints among Kaiser Permanente Southern California (KPSC) moderately-exposed members (n=1,373,419) in the week following the January 7, 2025 ignition of the LA Fires... 11

© 2025 Casey JA et al. JAMA Health Forum.

eMethods 1: Exposure assessment and confounding variables

Wildfire exposure: Kaiser Permanente Southern California's (KPSC) catchment area covers nine counties: Imperial, Kern, Los Angeles (LA), Orange, Riverside, San Bernardino, San Diego, San Luis Obispo, Santa Barbara, and Ventura. We classified census tracts in those counties *a priori* into three exposure levels based on their proximity to the maximum burn zone of seven LA-area wildfires as of January 17, 2025: Auto, Eaton, Hurst, Kenneth, Lidia, Palisades, and Sunset.[1] Highly exposed members resided in census tracts located <20km from a wildfire burn zone, moderately exposed members lived in tracts ≥20km away but within LA County, and minimally exposed members lived in tracts ≥20km away and in non-LA KPSC catchment counties (Imperial, Kern, Orange, Riverside, San Bernardino, San Diego, San Luis Obispo, Santa Barbara, and Ventura).

We opted to use proximity to estimate exposure, rather than other measures, such as estimated wildfire fine particulate matter ($PM_{2.5}$) concentrations or evacuation status, for several reasons. First, we were not interested in the effect of wildfire $PM_{2.5}$ alone, but rather the combined impact of air pollution, psychosocial stress, and community disruption brought on by the LA Fires.[2] We examined $PM_{2.5}$ concentrations in the KPSC catchment using daily data from US Environmental Protection Agency monitoring stations before finalizing our exposure assignment strategy. Between January 8-12, we observed elevated $PM_{2.5}$ concentrations across monitoring stations, with all elevated readings occurring at monitors in LA County. While concentrations were notably higher during this period, daily averages remained below 100 $\mu g/m^3$ at all monitoring stations. Second, we opted to use a strong causal inference design: two-stage interrupted time series modeling,[3] which uses ecological data and requires running analyses in groups. We coupled this approach with machine learning algorithms to better fit the data. Defining exposure based on proximity enabled us to quickly identify highly, moderately, and minimally exposed tracts in order to initiate analysis, which was useful for the rapid response nature of this study. Thus, due to our goal to capture a range of exposure pathways linking the LA Fires to healthcare utilization, study design, and rapid response, we defined exposure using proximity to wildfire burn zones. We buffered wildfire burn zones by 20km to identify highly exposed census tracts, as this area often overlapped with evacuation zones, and because wildfire $PM_{2.5}$, psychosocial stress, and disruption can extend beyond burn and evacuation zones. We considered all of LA County as moderately exposed due to elevated $PM_{2.5}$ concentrations across much of the county and substantial disruption there during the week following the LA Fires' ignition.

Meteorological variables: Time-varying census tract-level covariates included daily maximum and minimum temperature, precipitation, minimum and maximum humidity, wind velocity, and surface downward shortwave radiation processed from 4km^2 resolution gridMET data. We selected and controlled for these meteorological variables as they may operate as time-varying confounders in the relationship between wildfire exposure and cause-specific healthcare utilization.

Respiratory virus variables: We accessed weekly wastewater surveillance data on levels of three respiratory viruses— flu (influenza A and B), respiratory syncytial virus (RSV), and SARS-CoV-2— provided by the Los Angeles County Department of Public Health.[4] We controlled for these potential time-varying confounders in all models, but their impact was greatest in improving model fit for respiratory healthcare utilization.

eMethods 2: The 2-stage interrupted time-series design

This study uses an observational two-stage interrupted time series design using machine learning methods to estimate the number of visits attributable to wildfire exposure.[5] The first stage generates counterfactual trends of the expected number of visits for each of the exposure groups and type of encounter in the

© 2025 Casey JA et al. JAMA Health Forum.

absence of the wildfires using a hybrid Prophet-Extreme Gradient Boosting (XGBoost) model. We use the pre-wildfire period, November-January periods from November 1, 2022 to January 6, 2025, for training and testing of the modelling framework and apply the model to the post-wildfire ignition period (January 7, 2025 to January 20, 2025). In the second stage, we used the modelled estimates of the counterfactual trend to estimate excess visits from each level of wildfire exposure (highly, moderately, minimally).

Background on modeling framework

This machine learning approach combines the Prophet and XGBoost algorithms into a robust forecasting framework. First, Prophet decomposes the time series into trend, seasonality, and holiday components, which provides a structured representation of temporal patterns. Then, XGBoost augments this framework by modeling the residuals from the Prophet model through an ensemble of decision trees, minimizing the regularized objective function that balances prediction error and model complexity. The hybrid implementation leverages Prophet's strength in capturing explicit temporal patterns while using XGBoost's ability to identify complex relationships in the residuals and additional features.

Prophet is an additive regression model developed by Facebook for forecasting time series data. It decomposes time series into three main components: trend, seasonality, and holidays. The trend component is modeled using a logistic growth curve to accommodate saturated growth patterns. The holiday component allows for the incorporation of specific events or occasions that can influence the time series data.[6] The model can be expressed as:

$$y(t) = g(t) + s(t) + h(t) + \varepsilon_t$$

Where $g(t)$ represents the trend function, $s(t)$ is the seasonal component that captures periodic changes, $h(t)$ accounts for the effects of holidays, and ε_t is the error term.

XGBoost is an advanced implementation of gradient boosting, where predictive models are built as an ensemble of weak learners, typically decision trees. It operates by consecutively adding predictors to an ensemble, with each new predictor correcting its predecessor's errors through gradient descent optimization. XGBoost minimizes a regularized objective function that balances prediction error and model complexity, ensuring both accuracy and simplicity in the final model. The predictive model in XGBoost is formalized as follows:

$$\hat{y}_i = \sum_{k=1}^{K} f_k(x_i), \quad f_k \in \Phi$$

where $\hat{y}_i$ is the prediction for the i-th instance, x_i represents the features of the i-th instance, K is the number of trees in the model, f_k represents the individual decision trees, and Φ is the space of all possible regression trees.

Statistical modeling in this analysis

As we are using data from partial years (November-January) with strong weekend trends, we included indicators for U.S. federal holidays, time period (Nov 2022-Jan 2023, Nov 2023-Jan 2024, Nov 2024-Jan 2025), weekday/weekend, day of the week, month, and year in our model. We specifically removed certain holidays (Inauguration Day, Pulaski's Birthday, Juneteenth, and Decoration Memorial Day) and added an indicator for business closed holidays at Kaiser Permanente Southern California (Christmas Day, New Year's Day, Martin Luther King Day, Independence Day). We included covariates for daily maximum and minimum, precipitation, minimum and maximum relative humidity, wind velocity, and surface downward shortwave radiation from gridMET[7] as well as weekly wastewater surveillance data on levels of three respiratory viruses: flu, respiratory syncytial virus (RSV), and SARS-CoV-2[4] to improve

© 2025 Casey JA et al. JAMA Health Forum.

model predictions. Other time-invariant covariates that might influence healthcare utilization, such as socioeconomic or insurance status are inherently controlled by the ITS model, which compares places before and after the LA Fires to themselves. Due to differing seasonal patterns for respiratory virtual visits in Fall 2022 from the rest of the time series, models for these visits were restricted to January 2023 and later.

For our analysis, we used a time series split approach, dividing the dataset into training and testing periods. Specifically, we held out ~30% of pre-wildfire days for testing while using the remaining data for training. Within the training data, we implemented a time series cross-validation framework with 8 resampling slices using an assessment period ~20% of the training dataset for each fold. This approach maintains the temporal dependencies in the data while ensuring robust model validation.

We employed Prophet with XGBoost (Prophet Boost) using tunable parameters. For the XGBoost component, we tuned key parameters including number of variables per split (mtry: 2-10), minimum node size (min_n: 15-30), tree depth (3-8), learning rate (0.001-0.1), loss reduction (10^{-5} to 10), and early stopping iterations (10-50) for the XGBoost component. The tuning parameters for the XGBoost component were further specified within these ranges to find the best fit for some of the encounter types and causes. The tuning process used a space-filling design with 100 combinations.

We identified the optimal parameter set by minimizing the root mean square error (RMSE) across the cross-validation folds. We then used these optimal parameters to fit the final model on the entire training dataset and evaluated its performance on both the training data and the held-out test data using multiple metrics including mean absolute error (MAE), RMSE, mean absolute percentage error (MAPE), and R-squared.

We then used the optimal parameters to predict the number of visits for the whole study period. We employed a Monte Carlo simulation approach performing 1,000 model iterations to estimate 95% empirical confidence intervals (eCIs) for the predictions.[8] The eCIs were derived from the empirical distribution of the estimated number of visits for each day, taking the 2.5th and 97.5th percentiles as the lower and upper bounds.

To estimate the effect of the LA Fires for each encounter type, cause and wildfire exposure category, we calculated the difference between the observed and expected number of visits based on the model predictions. The number of excess visits was estimated for each day in the post-wildfire period for each encounter type, cause and exposure category. We estimated eCIs for excess visits by taking the difference between observed and the upper and lower bound of the prediction, which came from our Monte Carlo simulation. The total number of Kaiser beneficiaries in each exposure group was used to calculate the excess number of visits per 1,000 beneficiaries. A total number of visits was estimated by taking a sum of the excess visits and its upper and lower bounds across the week following the initial wildfires.

All statistical analyses were conducted with R software (version 4.4.1) using the *tidymodels tidyverse, modeltime, timetk, tictoc, metrics*, *fst* and *data.table* packages and Python version 3.12.2. The datasets and R scripts used in this study are publicly available on GitHub.

eMethods 3: Extrapolating excess visit counts to all insured LA County residents

We extrapolated excess visit counts for cardiovascular and respiratory-related virtual visits and respiratory-related outpatient visits in the combined highly- and moderately-exposed groups. To do so, we first enumerated individuals with health insurance in each census tract using data from the 2019–2023 American Community Survey.[9] We opted to extrapolate estimates only to the insured population because

© 2025 Casey JA et al. JAMA Health Forum.

KPSC requires insurance before providing outpatient or virtual care. We determined that KPSC members made up 13.6% of the highly-exposed insured population and 19.8% of the moderately-exposed insured population in LA County. Therefore, for cardiovascular and respiratory-related virtual visits and respiratory-related outpatient visits separately, we divided the estimated excess counts of visits in these categories for our highly-exposed group by 0.136 and our moderately-exposed group by 0.198, and then summed the counts by disease endpoint and care setting. This provided three simple estimates of excess total visits in LA County in the week following the LA Fires' ignition. This approach assumes that other LA residents had the same care-seeking behavior as KPSC members.

© 2025 Casey JA et al. JAMA Health Forum.

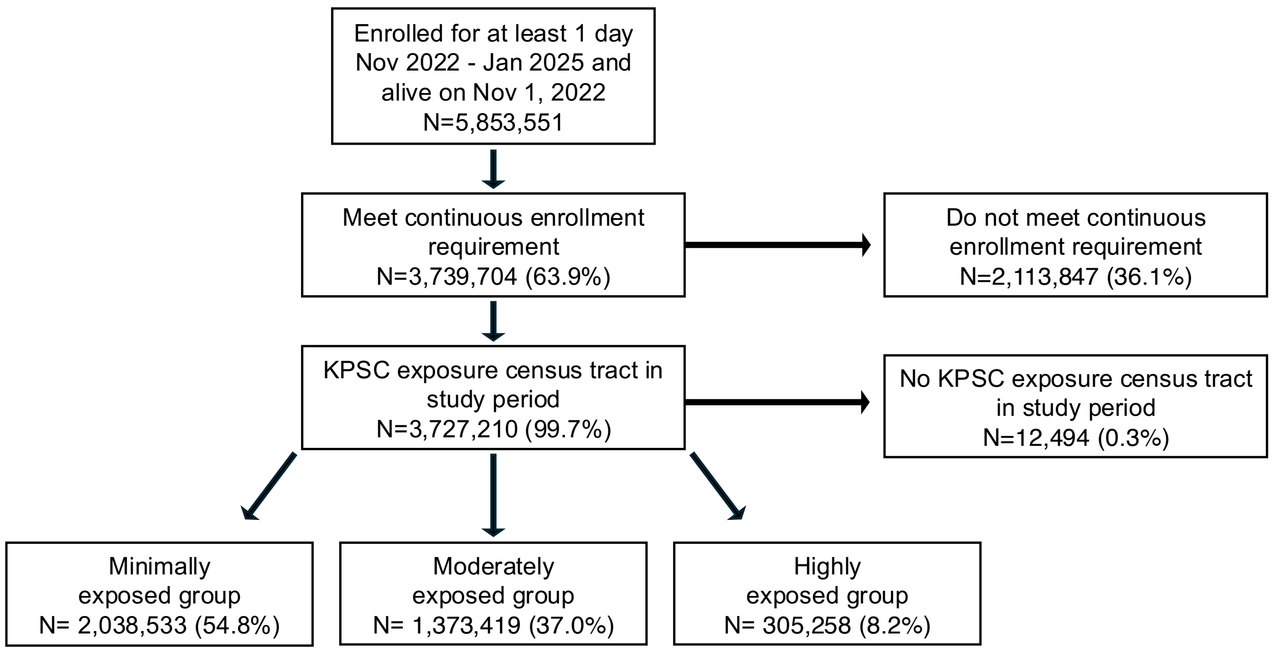

eFigure 1. Flow diagram depicting exclusion criteria for members of the KPSC study population, 2022-2025. To be included, KPSC members had to be enrolled from November to January 2022–2025, allowing 30-day enrollment gaps. The KPSC catchment counties included Imperial, Kern, Los Angeles, Orange, Riverside, San Bernardino, San Diego, San Luis Obispo, Santa Barbara, and Ventura. We excluded KPSC members who did not have a residential address in the KPSC catchment at baseline.
KPSC, Kaiser Permanente Southern California

© 2025 Casey JA et al. JAMA Health Forum.

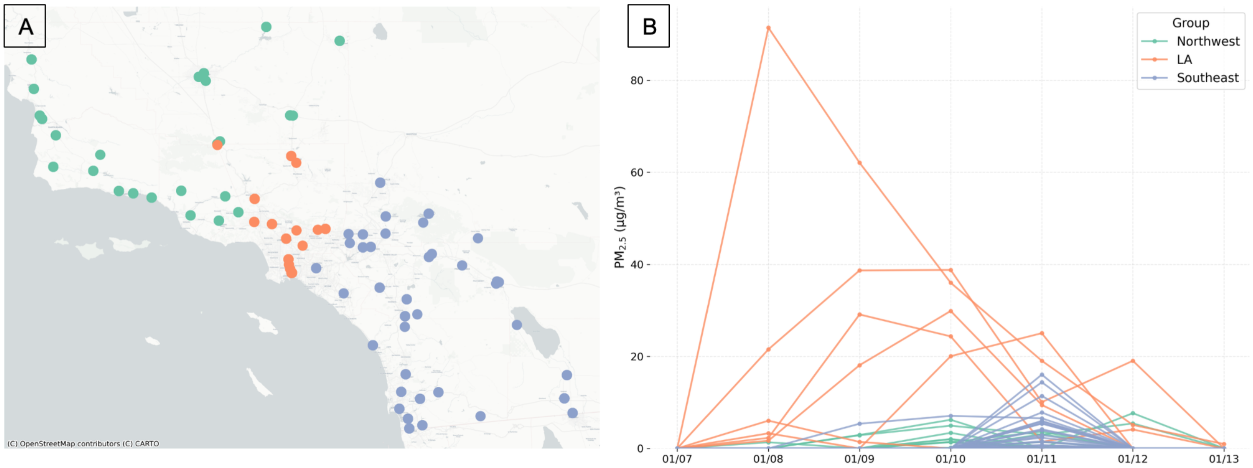

eFigure 2. (A) US Environmental Protection Agency monitoring station locations by geographic region, and (B) Station-level all-source daily $PM_{2.5}$ concentrations during the week following the LA Fires' ignition grouped by geographic region.

$PM_{2.5}$, Fine particulate matter

© 2025 Casey JA et al. JAMA Health Forum.

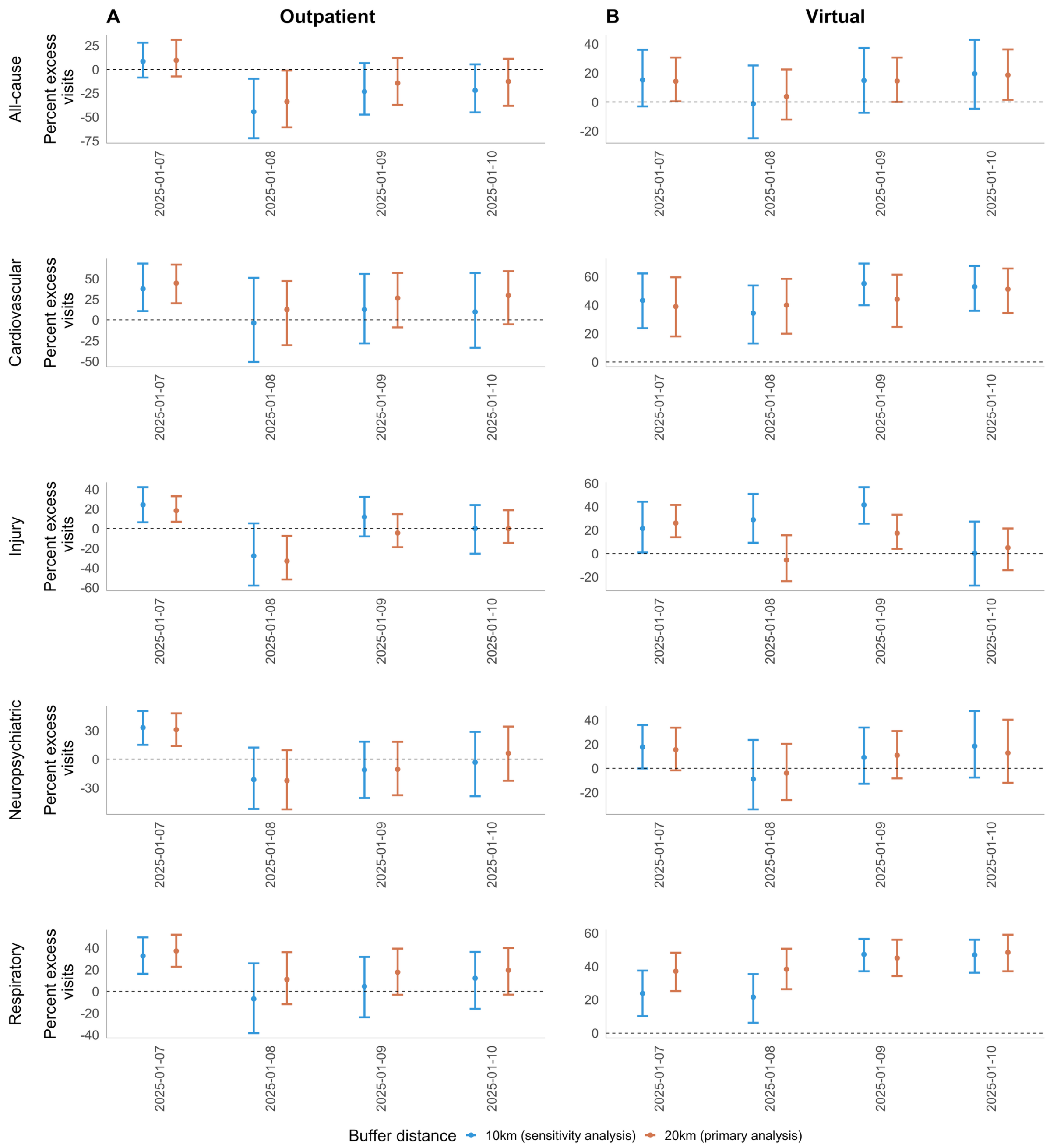

eFigure 3. Estimated change in the percent of outpatient and virtual acute care visits for all-cause, cardiovascular, injury, neuropsychiatric, and respiratory endpoints among Kaiser Permanente Southern California (KPSC) members residing in census tracts <10km (n= 114,579, sensitivity analysis) and <20km (n=305,258, primary analysis) of a burn zone in the week following the January 7, 2025 ignition of the LA Fires. We used the maximum wildfire burn zone reached by an LA or Ventura County wildfire as of January 16, 2025 to define exposure. In our sensitivity analysis, highly exposed members resided in a census tract located <10km burn zones. We also present results from the primary analysis where highly exposed members resided in a census tract located <20km burn zones. Results from an interrupted time series model using KPSC electronic health record data from November to January 2022–2025, with daily maximum and minimum temperature and humidity, wind velocity, and surface downward shortwave radiation and weekly wastewater surveillance data on levels of three respiratory viruses as covariates. We employed a Monte Carlo simulation approach, performing 1,000 model iterations to estimate 95% empirical confidence intervals (eCIs) for the predictions.

© 2025 Casey JA et al. JAMA Health Forum.

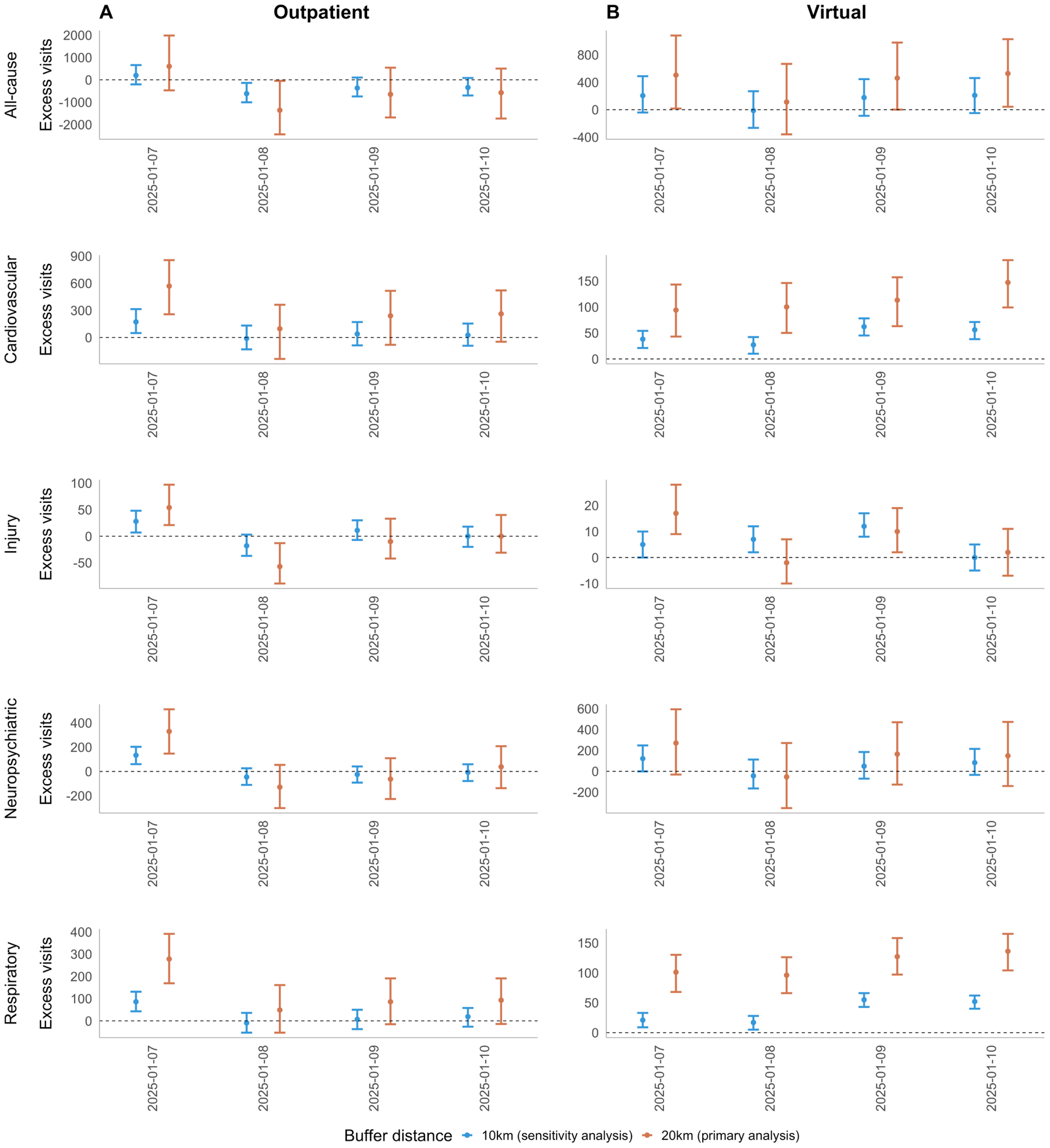

eFigure 4. Estimated change in the count of outpatient and virtual acute care visits for all-cause, cardiovascular, injury, neuropsychiatric, and respiratory endpoints among Kaiser Permanente Southern California (KPSC) members residing in census tracts <10km (n= 114,579, sensitivity analysis) and <20km (n=305,258, primary analysis) of a burn zone in the week following the January 7, 2025 ignition of the LA Fires. We used the maximum wildfire burn zone reached by an LA or Ventura County wildfire as of January 16, 2025 to define exposure. In our sensitivity analysis, highly exposed members resided in a census tract located <10km burn zones. We also present results from the primary analysis where highly exposed members resided in a census tract located <20km burn zones. Results from an interrupted time series model using KPSC electronic health record data from November to January 2022–2025, with daily maximum and minimum temperature and humidity, wind velocity, and surface downward shortwave radiation and weekly wastewater surveillance data on levels of three respiratory viruses as covariates. We employed a Monte Carlo simulation approach, performing 1,000 model iterations to estimate 95% empirical confidence intervals (eCIs) for the predictions.

© 2025 Casey JA et al. JAMA Health Forum.

eTable 1. Estimated change in the percent and count of virtual acute care visits for all-cause, cardiovascular, injury, neuropsychiatric, and respiratory endpoints among Kaiser Permanente Southern California (KPSC) highly-exposed members (n=305,258) in the week following the January 7, 2025 ignition of the LA Fires.

Excess percent (95% eCI)<br>*Excess count (95% eCI)*						
**Date**	**Weekday**	**All-cause**	**Cardiovascular**	**Injury**	**Neuropsychiatric**	**Respiratory**
2025-01-07	Tuesday	14% (0%, 31%)<br>504 (17, 1081)	39% (18%, 60%)<br>94 (43, 143)	26% (14%, 42%)<br>17 (9, 28)	15% (-2%, 34%)<br>271 (-31, 594)	36% (20%, 51%)<br>98 (55, 137)
2025-01-08	Wednesday	4% (-12%, 22%)<br>112 (-359, 668)	40% (20%, 58%)<br>100 (50, 146)	-6% (-24%, 16%)<br>-2 (-10, 7)	-4% (-26%, 20%)<br>-53 (-352, 271)	38% (22%, 54%)<br>94 (56, 134)
2025-01-09	Thursday	14% (0%, 31%)<br>461 (2, 978)	44% (25%, 62%)<br>113 (63, 157)	17% (4%, 33%)<br>10 (2, 19)	11% (-8%, 31%)<br>165 (-127, 470)	44% (30%, 58%)<br>125 (86, 164)
2025-01-10	Friday	19% (2%, 36%)<br>527 (43, 1028)	51% (34%, 66%)<br>147 (99, 190)	5% (-14%, 21%)<br>2 (-7, 11)	13% (-12%, 40%)<br>149 (-141, 473)	48% (34%, 62%)<br>133 (96, 173)
2025-01-11	Saturday	46% (-36%, 151%)<br>251 (-197, 828)	52% (-270%, 328%)<br>8 (-40, 49)	42% (-30%, 115%)<br>5 (-4, 14)	23% (-81%, 131%)<br>64 (-225, 365)	52% (-7%, 108%)<br>35 (-5, 72)
2025-01-12	Sunday	84% (-58%, 262%)<br>273 (-189, 850)	89% (-449%, 583%)<br>8 (-40, 52)	-94% (-497%, 374%)<br>-2 (-10, 7)	52% (-154%, 298%)<br>77 (-228, 441)	50% (-11%, 102%)<br>33 (-8, 68)
2025-01-13	Monday	23% (9%, 40%)<br>769 (298, 1326)	43% (22%, 62%)<br>97 (50, 141)	32% (18%, 48%)<br>20 (11, 30)	21% (1%, 44%)<br>328 (20, 688)	39% (25%, 52%)<br>102 (64, 137)
Weekly change		17% (-2%, 40%)<br>2896 (-385, 6759)	44% (17%, 68%)<br>567 (224, 879)	17% (-3%, 39%)<br>50 (-8, 115)	13% (-14%, 42%)<br>1002 (-1084, 3302)	42% (23%, 60%)<br>621 (346, 885)

We used the maximum wildfire burn zone reached by an LA or Ventura County wildfire as of January 16, 2025 to define exposure. Highly exposed members resided in a census tract located <20km burn zones. Results from an interrupted time series model using KPSC electronic health record data from November to January 2022–2025, with daily maximum and minimum temperature and humidity, wind velocity, and surface downward shortwave radiation and weekly wastewater surveillance data on levels of three respiratory viruses as covariates. We employed a Monte Carlo simulation approach, performing 1,000 model iterations to estimate 95% empirical confidence intervals (eCIs) for the predictions.

© 2025 Casey JA et al. JAMA Health Forum.

eTable 2. Estimated change in the percent and count of virtual acute care visits for all-cause, cardiovascular, injury, neuropsychiatric, and respiratory endpoints among Kaiser Permanente Southern California (KPSC) moderately-exposed members (n=1,373,419) in the week following the January 7, 2025 ignition of the LA Fires.

Excess percent (95% eCI)<br>*Excess count (95% eCI)*						
**Date**	**Weekday**	**All-cause**	**Cardiovascular**	**Injury**	**Neuropsychiatric**	**Respiratory**
2025-01-07	Tuesday	10% (-4%, 22%)<br>1286 (-520, 3018)	35% (12%, 56%)<br>397 (141, 633)	18% (5%, 32%)<br>42 (11, 75)	14% (2%, 28%)<br>871 (97, 1655)	31% (16%, 46%)<br>435 (225, 637)
2025-01-08	Wednesday	12% (-2%, 25%)<br>1490 (-194, 3299)	42% (21%, 62%)<br>501 (249, 740)	11% (-4%, 30%)<br>22 (-8, 59)	12% (-2%, 28%)<br>667 (-92, 1557)	37% (23%, 52%)<br>518 (319, 722)
2025-01-09	Thursday	17% (5%, 31%)<br>2277 (663, 4048)	39% (17%, 60%)<br>441 (193, 674)	13% (-2%, 31%)<br>26 (-5, 62)	19% (7%, 34%)<br>1100 (391, 1979)	38% (23%, 52%)<br>528 (325, 729)
2025-01-10	Friday	19% (4%, 37%)<br>2078 (480, 4078)	43% (19%, 66%)<br>469 (207, 709)	23% (7%, 42%)<br>47 (14, 85)	22% (6%, 42%)<br>997 (257, 1870)	39% (24%, 55%)<br>492 (298, 696)
2025-01-11	Saturday	46% (-18%, 116%)<br>1214 (-460, 3043)	42% (-200%, 262%)<br>45 (-213, 281)	68% (-3%, 149%)<br>29 (-1, 64)	24% (-36%, 96%)<br>277 (-411, 1097)	35% (-14%, 86%)<br>133 (-55, 329)
2025-01-12	Sunday	74% (-30%, 205%)<br>1179 (-489, 3287)	78% (-541%, 703%)<br>33 (-233, 302)	78% (-45%, 225%)<br>20 (-12, 58)	37% (-90%, 194%)<br>210 (-510, 1106)	30% (-18%, 76%)<br>105 (-64, 269)
2025-01-13	Monday	26% (14%, 41%)<br>3514 (1877, 5546)	40% (16%, 66%)<br>412 (162, 680)	28% (14%, 46%)<br>60 (29, 99)	24% (11%, 42%)<br>1344 (597, 2290)	38% (26%, 50%)<br>543 (372, 704)
Weekly change		19% (2%, 38%)<br>13038 (1358, 26319)	40% (9%, 70%)<br>2299 (506, 4019)	22% (3%, 45%)<br>248 (29, 503)	19% (1%, 40%)<br>5464 (329, 11554)	36% (19%, 54%)<br>2754 (1420, 4085)

We used the maximum wildfire burn zone reached by an LA or Ventura County wildfire as of January 16, 2025 to define exposure. Moderately exposed members lived in tracts ≥20km from a burn zone but within LA County. Results from an interrupted time series model using KPSC electronic health record data from November to January 2022–2025, with daily maximum and minimum temperature and humidity, wind velocity, and surface downward shortwave radiation and weekly wastewater surveillance data on levels of three respiratory viruses as covariates. We employed a Monte Carlo simulation approach, performing 1,000 model iterations to estimate 95% empirical confidence intervals (eCIs) for the predictions.

© 2025 Casey JA et al. JAMA Health Forum.

eTable 3. Estimated change in the percent and count of outpatient acute care visits for all-cause, cardiovascular, injury, neuropsychiatric, and respiratory endpoints among Kaiser Permanente Southern California (KPSC) highly-exposed members (n=305,258) in the week following the January 7, 2025 ignition of the LA Fires.

Excess percent (95% eCI)<br>Excess count (95% eCI)						
**Date**	**Weekday**	**All-cause**	**Cardiovascular**	**Injury**	**Neuropsychiatric**	**Respiratory**
2025-01-07	Tuesday	10% (-7%, 31%)<br>605 (-471, 1982)	44% (20%, 67%)<br>569 (257, 856)	18% (7%, 33%)<br>54 (21, 97)	31% (14%, 48%)<br>330 (147, 512)	37% (23%, 52%)<br>278 (169, 391)
2025-01-08	Wednesday	-34% (-61%, -1%)<br>-1361 (-2439, -48)	12% (-31%, 47%)<br>97 (-237, 362)	-33% (-52%, -7%)<br>-57 (-89, -13)	-22% (-53%, 9%)<br>-129 (-302, 54)	11% (-12%, 36%)<br>49 (-53, 161)
2025-01-09	Thursday	-14% (-37%, 12%)<br>-650 (-1684, 544)	26% (-9%, 57%)<br>240 (-81, 516)	-4% (-19%, 15%)<br>-10 (-42, 33)	-11% (-38%, 18%)<br>-63 (-227, 109)	18% (-3%, 39%)<br>86 (-15, 191)
2025-01-10	Friday	-13% (-38%, 11%)<br>-575 (-1732, 503)	30% (-5%, 59%)<br>262 (-47, 521)	0% (-15%, 19%)<br>0 (-31, 40)	6% (-23%, 34%)<br>38 (-138, 208)	19% (-3%, 40%)<br>93 (-14, 191)
2025-01-11	Saturday	11% (-151%, 192%)<br>73 (-1012, 1283)	-76% (-505%, 278%)<br>-54 (-359, 198)	-9% (-133%, 150%)<br>-3 (-38, 44)	-73% (-320%, 205%)<br>-48 (-208, 133)	-154% (-579%, 236%)<br>-40 (-151, 61)
2025-01-12	Sunday	268% (-912%, 1654%)<br>257 (-876, 1587)	-483% (-2889%, 1552%)<br>-63 (-376, 202)	-279% (-1392%, 1581%)<br>-8 (-42, 47)	-136% (-1036%, 875%)<br>-26 (-197, 166)	-183% (-784%, 447%)<br>-31 (-133, 76)
2025-01-13	Monday	18% (-1%, 40%)<br>1045 (-70, 2372)	47% (20%, 70%)<br>570 (242, 850)	13% (-1%, 35%)<br>31 (-2, 84)	29% (9%, 50%)<br>254 (78, 439)	47% (33%, 62%)<br>343 (240, 450)
Weekly change		-2% (-32%, 32%)<br>-606 (-8283, 8223)	32% (-12%, 68%)<br>1620 (-601, 3505)	1% (-19%, 28%)<br>8 (-223, 332)	9% (-22%, 42%)<br>356 (-847, 1621)	26% (2%, 52%)<br>777 (43, 1522)

We used the maximum wildfire burn zone reached by an LA or Ventura County wildfire as of January 16, 2025 to define exposure. Highly exposed members resided in a census tract located <20km burn zones. Results from an interrupted time series model using KPSC electronic health record data from November to January 2022–2025, with daily maximum and minimum temperature and humidity, wind velocity, and surface downward shortwave radiation and weekly wastewater surveillance data on levels of three respiratory viruses as covariates. We employed a Monte Carlo simulation approach, performing 1,000 model iterations to estimate 95% empirical confidence intervals (eCIs) for the predictions.

© 2025 Casey JA et al. JAMA Health Forum.

eTable 4. Estimated change in the percent and count of outpatient acute care visits for all-cause, cardiovascular, injury, neuropsychiatric, and respiratory endpoints among Kaiser Permanente Southern California (KPSC) moderately-exposed members (n=1,373,419) in the week following the January 7, 2025 ignition of the LA Fires.

		Excess percent (95% eCI)<br>*Excess count (95% eCI)*				
**Date**	**Weekday**	**All-cause**	**Cardiovascular**	**Injury**	**Neuropsychiatric**	**Respiratory**
2025-01-07	Tuesday	6% (-9%, 22%)<br>1443 (-2360, 5762)	38% (15%, 62%)<br>2022 (786, 3306)	9% (-4%, 24%)<br>102 (-40, 267)	28% (13%, 41%)<br>1263 (611, 1888)	33% (21%, 42%)<br>1024 (649, 1314)
2025-01-08	Wednesday	-8% (-25%, 16%)<br>-1605 (-5383, 3317)	32% (2%, 62%)<br>1388 (85, 2668)	-9% (-26%, 12%)<br>-79 (-223, 100)	13% (-6%, 31%)<br>453 (-231, 1105)	28% (12%, 40%)<br>696 (302, 1000)
2025-01-09	Thursday	5% (-12%, 26%)<br>1138 (-2724, 5843)	37% (9%, 63%)<br>1687 (404, 2903)	5% (-10%, 23%)<br>49 (-98, 222)	19% (1%, 35%)<br>681 (47, 1298)	29% (14%, 40%)<br>745 (353, 1042)
2025-01-10	Friday	5% (-13%, 27%)<br>1172 (-2852, 6182)	35% (4%, 67%)<br>1477 (178, 2807)	11% (-3%, 31%)<br>113 (-31, 304)	26% (8%, 48%)<br>896 (283, 1625)	30% (15%, 43%)<br>736 (367, 1049)
2025-01-11	Saturday	21% (-131%, 228%)<br>535 (-3296, 5721)	-138% (-663%, 429%)<br>-303 (-1452, 939)	-6% (-226%, 295%)<br>-4 (-147, 192)	-26% (-178%, 142%)<br>-106 (-727, 578)	-20% (-221%, 162%)<br>-34 (-373, 273)
2025-01-12	Sunday	189% (-740%, 1446%)<br>820 (-3217, 6290)	-1016% (-5152%, 3962%)<br>-295 (-1494, 1149)	-131% (-1024%, 1124%)<br>-21 (-164, 180)	-159% (-962%, 769%)<br>-127 (-769, 615)	-92% (-619%, 401%)<br>-64 (-433, 281)
2025-01-13	Monday	21% (5%, 42%)<br>5502 (1265, 11028)	41% (16%, 70%)<br>2042 (771, 3466)	23% (9%, 41%)<br>256 (103, 452)	33% (16%, 50%)<br>1387 (702, 2117)	42% (30%, 54%)<br>1288 (937, 1644)
Weekly change		7% (-15%, 36%)<br>9006 (-18567, 44142)	34% (-3%, 73%)<br>8018 (-722, 17238)	8% (-12%, 34%)<br>416 (-600, 1715)	22% (-0%, 46%)<br>4447 (-85, 9226)	31% (13%, 47%)<br>4389 (1801, 6603)

We used the maximum wildfire burn zone reached by an LA or Ventura County wildfire as of January 16, 2025 to define exposure. Moderately exposed members lived in tracts ≥20km from a burn zone but within LA County. Results from an interrupted time series model using KPSC electronic health record data from November to January 2022–2025, with daily maximum and minimum temperature and humidity, wind velocity, and surface downward shortwave radiation and weekly wastewater surveillance data on levels of three respiratory viruses as covariates. We employed a Monte Carlo simulation approach, performing 1,000 model iterations to estimate 95% empirical confidence intervals (eCIs) for the predictions.

© 2025 Casey JA et al. JAMA Health Forum.

References

1. CALFIRE. Current Emergency Incidents. https://www.fire.ca.gov/Incidents. Published 2025. Accessed 19 Jan 2025.
2. Benmarhnia T, Errett NA, Casey JA. Beneath the smoke: Understanding the public health impacts of the Los Angeles urban wildfires. *Environmental Epidemiology.* 2025;9(3):e388.
3. Nianogo RA, Benmarhnia T, O'Neill S. A comparison of quasi-experimental methods with data before and after an intervention: an introduction for epidemiologists and a simulation study. *International Journal of Epidemiology.* 2023;52(5):1522-1533.
4. Los Angeles County Department of Public Health. RESPWatch: Respiratory Illness Surveillance. http://ph.lacounty.gov/acd/respwatch/#Viruses. Accessed 02/12/2025.
5. Dey A, Ma Y, Carrasco-Escobar G, Han C, Rerolle F, Benmarhnia T. Two-stage interrupted time series analysis with machine learning: Evaluating the health effects of the 2018 wildfire smoke event in San Francisco County as a case study. *Am J Epidemiol.* 2025.
6. Taylor SJ, Letham B. Forecasting at scale. *The American Statistician.* 2018;72(1):37-45.
7. Abatzoglou JT. Development of gridded surface meteorological data for ecological applications and modelling. *International Journal of Climatology.* 2013;33(1):121-131.
8. Buckland ST. Monte Carlo Confidence Intervals. *Biometrics.* 1984;40(3):811-817.
9. Manson S, Schroeder J, Riper DV, Ruggles S. IPUMS National Historical Geographic Information System: Version 13.0 [Database]. University of Minnesota. http://doi.org/10.18128/D050.V12.0. Published 2023. Accessed 10 Oct 2020.

© 2025 Casey JA et al. JAMA Health Forum.
