# Supplementary material for: The 2025 Los Angeles Wildfires and Outpatient Acute Health Care Utilization
Source: JAMA Health Forum. 2025 Nov 26;6(11):e254632. doi: 10.1001/jamahealthforum.2025.4632 (PMC12658665; doi:10.1001/jamahealthforum.2025.4632)
Supplement: Supplement 2. — Data Sharing Statement [file jamahealthforum-e254632-s002.pdf]

Data Sharing Statement

Casey. The 2025 Los Angeles Wildfires and Outpatient Acute Health Care Utilization. *JAMA Health Forum*. Published November 26, 2025. doi:10.1001/jamahealthforum.2025.4632

Data
Data available: No

Additional Information
Explanation for why data not available: Anonymized data that support the findings of this study may be made available from the investigative team in the following conditions: (1) agreement to collaborate with the study team on all publications, (2) provision of external funding for administrative and investigator time necessary for this collaboration, (3) demonstration that the external investigative team is qualified and has documented evidence of training for human subjects protections, and (4) agreement to abide by the terms outlined in data use agreements between institutions. Relevant code is publicly available via GitHub: https://github.com/heathermcb/los_angeles_2025_fire_disasters.
